# Mitoepigenetics and Its Emerging Roles in Cancer

**DOI:** 10.3389/fcell.2020.00004

**Published:** 2020-01-23

**Authors:** Zhen Dong, Longjun Pu, Hongjuan Cui

**Affiliations:** ^1^State Key Laboratory of Silkworm Genome Biology, Institute of Sericulture and Systems Biology, Southwest University, Chongqing, China; ^2^Cancer Center, Medical Research Institute, Southwest University, Chongqing, China; ^3^Engineering Research Center for Cancer Biomedical and Translational Medicine, Southwest University, Chongqing, China; ^4^Chongqing Engineering and Technology Research Center for Silk Biomaterials and Regenerative Medicine, Southwest University, Chongqing, China; ^5^Umeå Centre for Molecular Medicine, Umeå University, Umeå, Sweden

**Keywords:** mtDNA methylation, mitoepigenetics, mtRNA modification, non-coding RNAs, cancer

## Abstract

In human beings, there is a ∼16,569 bp circular mitochondrial DNA (mtDNA) encoding 22 tRNAs, 12S and 16S rRNAs, 13 polypeptides that constitute the central core of ETC/OxPhos complexes, and some non-coding RNAs. Recently, mtDNA has been shown to have some covalent modifications such as methylation or hydroxylmethylation, which play pivotal epigenetic roles in mtDNA replication and transcription. Post-translational modifications of proteins in mitochondrial nucleoids such as mitochondrial transcription factor A (TFAM) also emerge as essential epigenetic modulations in mtDNA replication and transcription. Post-transcriptional modifications of mitochondrial RNAs (mtRNAs) including mt-rRNAs, mt-tRNAs and mt-mRNAs are important epigenetic modulations. Besides, mtDNA or nuclear DNA (n-DNA)-derived non-coding RNAs also play important roles in the regulation of translation and function of mitochondrial genes. These evidences introduce a novel concept of mitoepigenetics that refers to the study of modulations in the mitochondria that alter heritable phenotype in mitochondria itself without changing the mtDNA sequence. Since mitochondrial dysfunction contributes to carcinogenesis and tumor development, mitoepigenetics is also essential for cancer. Understanding the mode of actions of mitoepigenetics in cancers may shade light on the clinical diagnosis and prevention of these diseases. In this review, we summarize the present study about modifications in mtDNA, mtRNA and nucleoids and modulations of mtDNA/nDNA-derived non-coding RNAs that affect mtDNA translation/function, and overview recent studies of mitoepigenetic alterations in cancer.

## Introduction

Epigenetics is the study of mitotically and/or meiotically heritable phenotype alterations that do not entail a change in DNA sequence (Wu and Morris, 2001). Epigenetics in nuclear genome (nDNA) has been well described and characterized. Generally, epigenetic regulation contains three levels of biological actions, including covalent modifications in DNA bases, histone variants, post-translational modifications of histones, RNA modifications, and non-coding RNA (ncRNA) modulations (Dupont et al., 2009; Peschansky and Wahlestedt, 2014). Epigenetic regulation has been shown to be an important biological process that participates in tumorigenesis and cancer development, and epigenetic biomarkers or targets can be used in diagnosis, prognosis, and treatment of these diseases (Kondo et al., 2017; Dong and Cui, 2018; Nebbioso et al., 2018; Zhu et al., 2019).

As cellular organelles present in almost all eukaryotic cells, mitochondria are places where ATP is biosynthesized and are essential for various cellular biological processes, including reactive oxygen species (ROS) generation, intracellular Ca^2+^ signaling, heme metabolism, intrinsic apoptosis, mitophagy, metabolism and cell cycle progression (Yien et al., 2014; Picard et al., 2016; van der Bliek et al., 2017; Gómez-Durán et al., 2018). Genetic mutations in mitochondrial DNA (mtDNA) and perturbations in mitochondrial proteins can cause dysfunction of mitochondria that has been shown to be tightly associated with many mitochondrial diseases and cancer (Taylor and Turnbull, 2005; Garone et al., 2012a, b, 2013; Gómez-Durán et al., 2012; Ronchi et al., 2012; Kullar et al., 2017; Chinnery and Gómez-Durán, 2018; Garone and Viscomi, 2018; Andreazza et al., 2019). Therefore, mitochondria are promising targets for the treatment of these diseases (Gómez-Durán et al., 2010; Zong et al., 2016; Dong et al., 2019). Recently, in addition to mitogenetics, epigenetics in mitochondria (mitoepigenetics) also emerges as an important regulatory mode that is related to human physiology and disorders, such as stemness, drug addiction, neurodegenerative diseases, cardiovascular diseases and metabolic diseases (Wallace, 1992; Gómez-Durán et al., 2010; Manev and Dzitoyeva, 2013; Kalani et al., 2014; Sadakierska-Chudy et al., 2014; Ghosh et al., 2015; Gao D. et al., 2017; Stimpfel et al., 2018; Coppede and Stoccoro, 2019). Importantly, this kind of mode of action has also emerged to be associated with cancers (Ferreira et al., 2015; Lozano-Rosas et al., 2018). However, mitoepigenetics has not been well depicted.

Herein, we define the concept of mitoepigenetics as the study of modulations occurring in the mitochondria that induce heritable phenotype alterations in mitochondria without involving a change of mtDNA sequence. Based on current studies, mitoepigenetics comprises of four levels: mtDNA methylation/hydroxylmethylation, mitochondrial nucleoid modifications, mtRNA modifications, and mtDNA-derived or nDNA-derived non-coding RNA modulations during mtDNA-encoded gene translation/function. In this article, we review the current study on mitoepigenetics and its roles in cancers, so as to provide a new sight for the diagnosis and treatment of these disorders.

## mtDNA and its Modifications

### mtDNA

**FIGURE 1:**
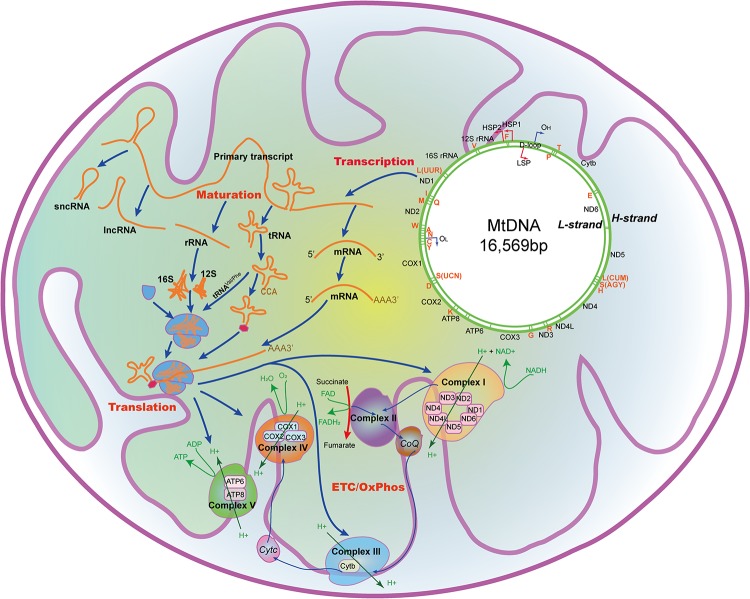
The mtDNA and the processing and function of its encoding genes in the mitochondria. mtDNA with 16,569 nucleotides encodes 22 tRNAs, 2 rRNAs, 13 peptides that constitutes the ETC/OxPhos, and some non-coding RNAs. A, mt-tRNA^Ala^; C, mt-tRNA^Cys^; D, mt-tRNA^Asp^; E, mt-tRNA^Glu^; F, mt-tRNA^Phe^; G, mt-tRNA^Gly^; H, mt-tRNA^His^; I, mt-tRNA^Ile^; K, mt-tRNA^Lys^; L(CUN), mt-tRNA^Leu(CUN)^; L(UUR), mt-tRNA^Leu(UUR)^; M, mt-tRNA^Met^; N, mt-tRNA^Asn^; P, mt-tRNA^Pro^; Q, mt-tRNA^Gln^; R, mt-tRNA^Arg^; S(AGY), mt-tRNA^Ser(AGY)^; S(UCN), mt-tRNA^Ser(UCN)^; T, mt-tRNA^Thr^; V, mt-tRNA^Val^; W, mt-tRNA^Trp^; Y, mt-tRNA^Tyr^; ATP6/8, Mitochondrially encoded ATP synthase membrane subunit 6/8; CoQ, Coenzyme Q; COX1/2/3, Mitochondrially encoded cytochrome C oxidase I/II/III; Cytb, Cytochrome b; Cytc, Cytochrome c; ETC, Electron transport chain; FAD, Flavine adenine dinucleotide; FADH_2_, Flavine adenine dinucleotide, reduced; HSP1/2, H-strand promotor 1/2; H-strand, Heavy strand; LSP, L-strand promotor; L-strand, Light strand; NAD^+^, Nicotinamide adenine dinucleotide; NADH, Nicotinamide adenine dinucleotide, reduced; ND1/2/3/4/4L/5/6, Mitochondrially encoded NADH:ubiquinone oxidoreductase core subunit 1/2/3/4/4L/5/6; O_L_, L-strand origin of replication; O_H_, H-strand origin of replication; OxPhos, Oxidative phosphorylation.

mtDNA exists in the matrix or the inner membrane of mitochondria. Genome signature comparisons reveal that mtDNA is analogous to prokaryotic genome (Campbell et al., 1999). Especially, alpha-proteobacteria seems to be the most likely bacterial ancestor of the mitochondria (Gray, 2012; Muñoz-Gómez et al., 2017; Martijn et al., 2018). mtDNA has a loop structure of guanine-rich heavy chains (H-strand) and cytosine-rich light chains (L-strand) (Chinnery and Hudson, 2013). The length of mtDNA ranges from 15,000 to 17,000 bp in different species (Chinnery and Hudson, 2013). Human mtDNA is a ∼16,569 bp circular DNA that encodes 37 genes (28 on the H-strand and 9 on the L-strand), including 2 rRNAs (12S and 16S rRNAs), 22 tRNAs, and 13 proteins in the electron transport chain (ETC)/oxidative phosphorylation (OxPhos) system (Table 1 and Figure 1) (Andrews et al., 1999; Brandon et al., 2005; Chinnery and Hudson, 2013). Besides, mtDNA also contains some pseudogenes (Woischnik and Moraes, 2002; Gunbin et al., 2017) and encodes non-coding RNAs (ncRNAs), including long non-coding RNAs (lncRNAs) (Rackham et al., 2011) and small non-coding RNAs (sncRNAs), such as microRNAs (Lung et al., 2006; Barrey et al., 2011; Bandiera et al., 2013; Ro et al., 2013; Duarte et al., 2014). Mitochondrial genome has some unique genetic characteristics, including high mutation, heteroplasmy, threshold effect, maternal inheritance and mitotic segregation (Wallace, 2005). The copy number of mtDNA varies between 100 and 10 000 per cell dependent upon cellular energy demand (Srinivasan et al., 2017).

**TABLE 1:** Protein-coding and RNA genes that encoded by human mtDNA (Pseudogenes and non-coding RNA genes are not included in this table).

Gene symbol	Alternative names	Description	Location in mtDNA (H/L-strand)	Size (nt)
MT-RNR1	12S rRNA	Mitochondrially encoded 12S RNA	648/649–1601, H	954/955
MT-RNR2	16S rRNA	Mitochondrially encoded 16S RNA	1,671–3,229, H	1,559
MT-TL2	mt-tRNA^Leu(CUN)^	Mitochondrially Encoded TRNA-Leu (CUN) 2	12,266–12,336, H	71
MT-TI	mt-tRNA^Ile^	Mitochondrially Encoded TRNA-Ile (AUU/C)	4,263–4,331, H	69
MT-TQ	mt-tRNA^Gln^	Mitochondrially Encoded TRNA-Gln (CAA/G)	4,329–4,400, L	72
MT-TW	mt-tRNA^Trp^	Mitochondrially Encoded TRNA-Trp (UGA/G)	5,512–5,579, H	68
MT-TA	mt-tRNA^Ala^	Mitochondrially Encoded TRNA-Ala (GCN)	5,587–5,655, L	69
MT-TN	mt-tRNA^Asn^	Mitochondrially Encoded TRNA-Asn (AAU/C)	5,657–5,729, L	73
MT-TC	mt-tRNA^Cys^	Mitochondrially Encoded TRNA-Cys (UGU/C)	5,761–5,826, L	66
MT-TY	mt-tRNA^Tyr^	Mitochondrially Encoded TRNA-Tyr (UAU/C)	5,826–5,891, L	66
MT-TS2	mt-tRNA^Ser(AGY)^	Mitochondrially Encoded TRNA-Ser (AGU/C) 2	12,207–12,265, H	59
MT-TD	mt-tRNA^Asp^	Mitochondrially Encoded TRNA-Asp (GAU/C)	7,518–7,585, H	68
MT-TK	mt-tRNA^Lys^	Mitochondrially Encoded TRNA-Lys (AAA/G)	8,295–8,364, H	70
MT-TG	mt-tRNA^Gly^	Mitochondrially Encoded TRNA-Gly (GGN)	9,991–10,058, H	68
MT-TR	mt-tRNA^Arg^	Mitochondrially Encoded TRNA-Arg (CGN)	10,405–10,469, H	65
MT-TH	mt-tRNA^His^	Mitochondrially Encoded TRNA-His (CAU/C)	12,138–12,206, H	69
MT-TS1	mt-tRNA^Ser(UCN)^	Mitochondrially Encoded TRNA-Ser (UCN) 1	7,445–7,516, L	72
MT-TE	mt-tRNA^Glu^	Mitochondrially Encoded TRNA-Glu (GAA/G)	14,674–14,742, L	69
MT-TP	mt-tRNA^Pro^	Mitochondrially Encoded TRNA-Pro (CCN)	15,955–16,023, L	69
MT-TT	mt-tRNA^Thr^	Mitochondrially Encoded TRNA-Thr (ACN)	15,888–15,953, H	66
MT-TF	mt-tRNA^Phe^	Mitochondrially Encoded TRNA-Phe (UUU/C)	577–647, H	71
MT-TV	mt-tRNA^Val^	Mitochondrially Encoded TRNA-Val (GUN)	1,602–1,670, H	69
MT-TM	mt-tRNA^Met^	Mitochondrially Encoded TRNA-Met (AUA/G)	4,402–4,469, H	68
MT-TL1	mt-tRNA^Leu(UUR)^	Mitochondrially Encoded TRNA-Leu (UUA/G) 1	3,230–3,304, H	75
MT-CYB	Cytochrome b	Mitochondrially encoded cytochrome b	14,747–15,887, H	1,141
MT-CO1	COX 1	Mitochondrially Encoded Cytochrome C Oxidase I	5901–7442, H	1,542
MT-CO2	COX 2	Mitochondrially Encoded Cytochrome C Oxidase II	7586–8294, H	709
MT-CO3	COX 3	Mitochondrially Encoded Cytochrome C Oxidase III	9,207–9,990, H	784
MT-ND1	ND 1	Mitochondrially Encoded NADH:Ubiquinone Oxidoreductase Core Subunit 1	3,305–4,262, H	958
MT-ND2	ND 2	Mitochondrially Encoded NADH:Ubiquinone Oxidoreductase Core Subunit 2	4,470–5,511, H	1,042
MT-ND3	ND 3	Mitochondrially Encoded NADH:Ubiquinone Oxidoreductase Core Subunit 3	10,059–10,404, H	346
MT-ND4	ND 4	Mitochondrially Encoded NADH:Ubiquinone Oxidoreductase Core Subunit 4	10,760–12,137, H	1,378
MT-ND4L	ND 4L	Mitochondrially Encoded NADH:Ubiquinone Oxidoreductase Core Subunit 4L	10,470–10,766, H	297
MT-ND5	ND 5	Mitochondrially Encoded NADH:Ubiquinone Oxidoreductase Core Subunit 5	12,337–14,148, H	1,812
MT-ND6	ND 6	Mitochondrially Encoded NADH:Ubiquinone Oxidoreductase Core Subunit 6	14,149–14,673, L	525
MT-ATP6	ATPase 6	Mitochondrially Encoded ATP Synthase Membrane Subunit 6	8,527–9,206, H	681
MT-ATP8	ATPase 8	Mitochondrially Encoded ATP Synthase Membrane Subunit 8	8,365–8,572, H	207

Unlike nDNA, mtDNA contains only one non-coding triple-helical region (16,024-576, ∼1000 bp, ∼7% of all sequence), the displacement loop (D-loop) formed by abortive initiation of replication (Yamamoto, 2001). Both replication and transcription are initiated from D-loop, which contains the H/L-strand promotor (HSP1/LSP1), and the H-strand origin of replication (O_H_) (Jemt et al., 2015). Sixty bp upstream of HSP1, there is a HSP2. There are also some specific sites (IT_L_, IT_H__1_, IT_H__2_) within the promotors, where mtDNA transcription initiates. Mitochondrial RNAs are firstly transcribed as primary transcripts, which are subsequently cleaved by enzymes and affected by specific nucleotide modifications to yield polycistronic precursors and finally mature RNAs (Van Haute et al., 2015). Besides, like prokaryotic cells, mtDNA lacks intronic regions, and intergenic sequences are either absent or only a few nucleotide bases long. Some genes, such as MT-ATP6/8 and MT-ND4/4L, have overlapping regions.

### mtDNA Modifications

Although mitochondrial genome has a diminutive size, mutations in mtDNA occur frequently, because mtDNA lacks the error checking system that nDNA has. Some of mutations are tightly associated with inherited diseases (Taylor and Turnbull, 2005). Apart from mtDNA mutations, mtDNA is also shown to have some modifications, like that in nDNA.

**FIGURE 2:**
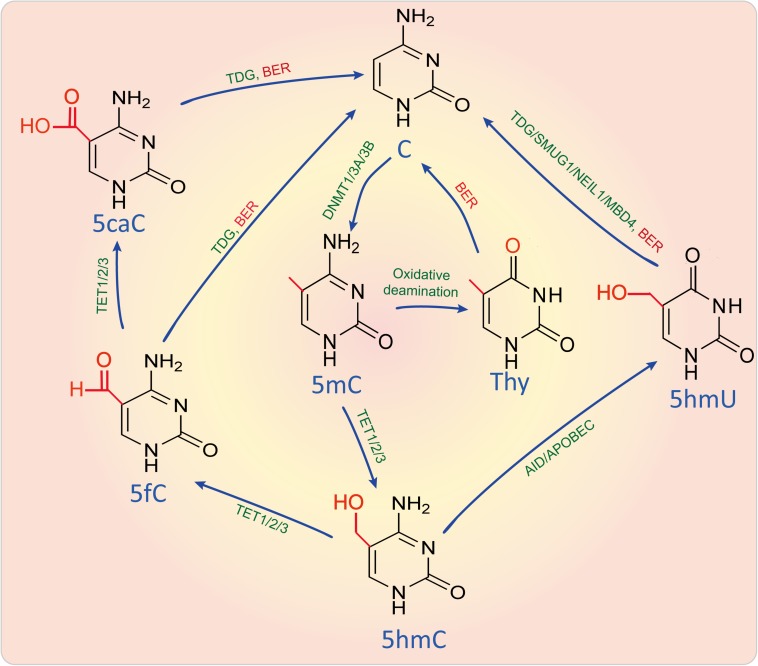
DNA methylation and active demethylation. DNA can be methylated by DNMTs and demethylated by active demethylation through oxidizing, deaminating and base-excision repair. Enzymes were marked in green, metabolites were marked in blue, while biological process like BER was marked in red. 5caC, 5-Carboxylcytosine; 5fC, 5-Formylcytosine; 5hmC, 5-Hydroxymethylcytosine; 5hmU, 5-Hydroxymethyluracil; 5-AID, activation induced cytidine deaminase; APOBEC, Apolipoprotein B mRNA editing enzyme catalytic subunit; BER, Base-excision repair; DNMT1/3A/3B, DNA methyltransferase 1/3A/3B; MBD4, Methyl-CpG binding domain 4, DNA glycosylase; NEIL1, Nei like DNA glycosylase 1; SMUG1, Single-strand-selective monofunctional uracil-DNA glycosylase 1; TET1/2/3, Tet methylcytosine dioxygenase 1/2/3; C, Cytosine; TDG, Thymine DNA glycosylase; Thy, Thymine.

As shown in Figure 2, DNA can be methylated by methyltransferases, which can transfer a methyl group from a methyl donor *S*-adenosylmethionine (SAM) onto the C5 position of the cytosine to form 5-methylcytosine (5mC) by the aid of DNA methyltransferases (DNMTs) such as DNMT1, DNMT3A, and DNMT3B (Moore et al., 2013). Methylation leads to changes of the molecular structure in DNA and affects transcription factor, RNA polymerase, topoisomerase, or inhibitory protein binding to DNA, resulting in dysfunction of gene transcription and expression (Moore et al., 2013). DNA 5mC can also be demethylated via either passive demethylation carried out by dilution by replication without *de novo* methylation or active demethylation carried out by oxidation or deamination.

During active demethylation pathway, some of 5mC sites can also be catalyzed and oxidized by 2-oxoglutarate and Fe(II)-dependent oxygenases of the ten-eleven-translocation (TET) proteins, including TET1, TET2, and TET3, to form 5-hydroxymethylcytosine (5hmC), which is considered as a possible intermediate in a replication-independent DNA demethylation pathway (Richa and Sinha, 2014). 5hmC is enriched in active genes that have a strong depletion of 5mC (Mellen et al., 2012). With the aid of TET1/2/3, 5hmC is further catalyzed into 5-formylcytosine (5fC) and 5-carboxylcytosine (5caC), which can be subsequently excised and replaced via base excision repair (BER). Besides, 5mC and 5hmC can also be deaminated to yield thymine and 5-hydroxymethyluracil (5hmU) by the aid of activation induced cytidine deaminase (AID)/apolipoprotein B mRNA editing enzyme and catalytic polypeptide (APOBEC). This results in a thymine-guanine mismatch that can lead to a DNA repair in which thymine and 5hmU can be replaced by unmethylated cytosine (Jang et al., 2017). However, 5hmC seems to be not only the intermediate of DNA demethylation, but is also a major element in the modulation of chromatin structure and gene expression through binding with methyl-CpG-binding protein 2 (MeCP2) (Mellen et al., 2012).

With the development of technology for detecting methylation, this kind of modification was also found in mtDNA. Distribution of 5mC seems to be conserved in mitochondrial genomes across all cell and tissue types (Ghosh et al., 2014). mtDNA methylation is usually found within the non-coding D-loop and gene start sites (GSS) (Mposhi et al., 2017), implying that methylation in mtDNA can affect mtDNA replication and transcription. Stimulating mtDNA replication results in increasing methylation (Rebelo et al., 2009), confirming that methylation can also be a feedback regulatory mode that maintains mtDNA copy number.

CpG dinucleotides are the most prominent regions where methylation occurs, however, non-CpG sites, such as CpA, CpT, and CpC also have methylations (Jang et al., 2017). The abundance of CpG sites varies in animal, fungal, protist, and plant mitochondrial genomes. Like nDNA, human mtDNA contains a relatively low frequency of CpG sites (435 in 16 659 nucleotides, 2.61%) (Cardon et al., 1994). Methylation of CpG in the H-strand promoter (HSP1) induces TFAM multimerization to augment cooperativity and enhances its binding affinity to mtDNA, compared to that of the non-methylated DNA. Although TFAM-dependent DNA compaction is not affected by methylation of CpG sites, transcription initiation from the three mitochondrial promoters is significantly impaired by CpG methylation (Dostal and Churchill, 2019). However, a study shows that mtDNA methylation mainly occurs within non-CpG sites of the promoter region of the H-strand, which is essential for mtDNA replication and transcription (Bellizzi et al., 2013).

5mC in mtDNA is catalyzed by mtDNMT1, an isoform of DNMT1. mtDNMT contains a mitochondrial targeting sequence, which can make it translocated into mitochondria (Shock et al., 2011). However, DNA methyltransferases seem to contribute to CpG methylation in the D-loop, while not non-CpG methylation (Bellizzi et al., 2013), because MtDNMT1 binding to the mtDNA is observed to be associated with the density of CpG sites (60).

5hmC is also found in mtDNA and it seems to promote demethylation through impairing mtDNMT1-mediated remethylation during replication (Manev and Dzitoyeva, 2013). However, the detailed process and the enzymes involved are not identified yet.

## Mitochondrial Nucleoid and its Modifications

### Mitochondrial Nucleoid

**FIGURE 3:**
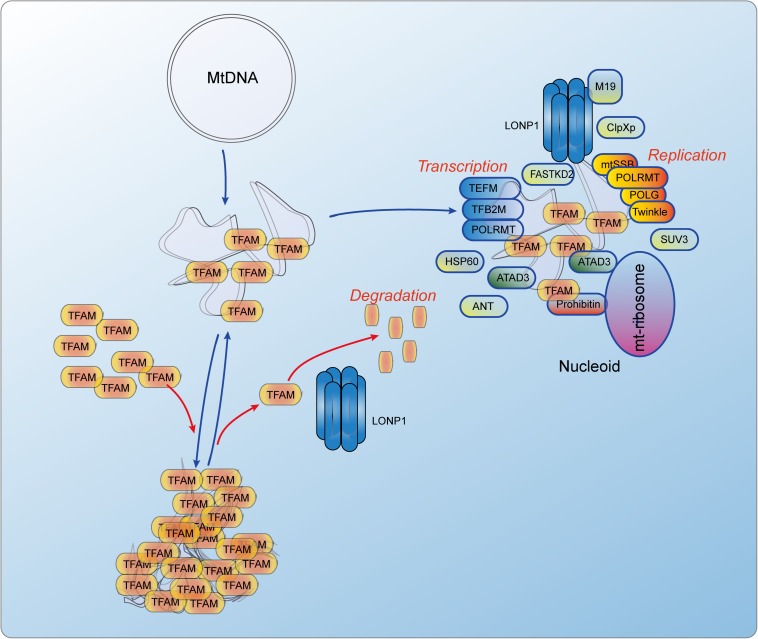
The dynamics of TFAM controlled mitochondrial nucleoid and the constitutions of nucleiod. TFAm can directly bind to mtDNA and functions as a histone-like protein. Its degradation is mediated by LONP1, an AAA + Lon protease. There are more than 50 nucleoid-associated proteins including POLRMT, TFAM, TFB2M and TEFM that initiate the mtDNA transcription, and POLG, Twinkle and mtSSB that initiate the mtDNA replication. There are also some proteins that are related to RNA processing and nucleoid regulation. ANT, Adenine nucleotide translocator; ATAD3, ATPase family AAA domain containing 3; ClpXp, ATP-dependent Clp protease ATP-binding subunit clpX-like, mitochondrial; FASTKD2, FAST kinase domains 2; HSP60, Short heat shock protein 60; LONP1, Lon peptidase 1, mitochondrial; M19, Mitochondrial protein M19; mtSSB, Single-stranded DNA binding protein 1, mitochondrial; POLG, DNA polymerase gamma; POLRMT, RNA polymerase mitochondrial; SUV3, ATP-dependent RNA helicase SUV3, mitochondrial; TEFM, Transcription elongation factor, mitochondrial; TFAM, Transcription factor A, mitochondrial; TFB2M, Transcription factor B2, mitochondrial.

Similarly to nDNA, mtDNA is also packed by proteins to form a protein-DNA structure referred to as a nucleoid. It is located in mitochondrial pseudocompartments, and some of its proteins may play histone-like architectural roles (Kanki et al., 2004). There are more than 50 nucleoid-associated proteins that can either temporarily or permanently associate with mtDNA or other nucleoid-associated proteins to maintain mtDNA and regulate gene expression (Figure 3). In nucleoid, mitochondrial transcription factor A (TFAM), mitochondrial polymerase γ (POLG), ATPase family AAA-domain-containing protein 3 (ATAD3), mitochondrial AAA protease (LONP1), and mitochondrial single-stranded DNA-binding protein (mtSSB) possibly directly interact with the D-loop region of mtDNA (Lee and Han, 2017). Mitochondrial RNA polymerase (POLRMT), TFAM, mitochondrial transcription factor B2 (TFB2M) and mitochondrial transcriptional elongation factor (TEFM) are key components of the mitochondrial transcription (Shokolenko and Alexeyev, 2017). POLG, Twinkle, mtSSB are key components of the mitochondrial replication (Young and Copeland, 2016). Besides them, nucleoid-associated proteins also include RNA helicases (e.g., SUV3), RNA-binding proteins (e.g., FASTKD2), quality-control proteases (e.g., lon-like peptidase LONP1 and caseionlytic peptidase CLPXP), as well as mitochondrial RNA processing proteins (Koc and Spremulli, 2003; Szczesny et al., 2013; Popow et al., 2015; Levytskyy et al., 2016; Lee and Han, 2017). These evidences suggest that nucleoid may be a place where mtRNAs are processed and mitoribosomes are assembled. Recently, post-transcriptional mtRNA processing and ribosome biogenesis including, mtRNA maturation, ribosome assembly, and translation initiation may occur within mitochondrial RNA granules (MRGs), dynamic structures that juxtapose to nucleoids (Jourdain et al., 2013; Antonicka and Shoubridge, 2015; Hensen et al., 2019). MRGs are transiently associated with active nucleoids where they are assembled around the newly synthesized primary transcripts, then MRGs become detached and locate within the inner mitochondrial matrix and subsequent events in mitochondrial gene expression take place (Jourdain et al., 2016).

TFAM is a member of the high-mobility group domain proteins family and can form a U-turn with an overall bend of 180° on unspecific mtDNA sequence, functioning as a transcription and packaging factor (Ngo et al., 2011). In mammalian cells, this protein is very abundant. Per each mtDNA molecule, there are about 1,000 molecules of TFAM protein, which means that there is a TFAM molecule in every 16 bp of mtDNA (Farge and Falkenberg, 2019). Importantly, only with TFAM nucleoid compaction of mtDNA can sufficiently complete (Kaufman et al., 2007). In addition, single TFAM protein can also bridge neighboring mtDNA duplexes to form a cross-strand binding and looping out (Kukat et al., 2015). In the condition of high TFAM/mtDNA ratio, the combination of duplex bending and cross-strand binding result into mtDNA full compaction, which leads to the blockade of mtDNA transcription and replication (Ngo et al., 2014). Besides, TFAM can also act as a homodimer, which promotes looping of the DNA (Ngo et al., 2014). In summary, TFAM is the only nucleoid-associated protein that can stringently fulfill the criteria of a true mtDNA packaging factor (Bonekamp and Larsson, 2018). Unbalanced levels (low or high) of TFAM result in decreasing mtDNA methylation (Rebelo et al., 2009).

Nucleoids have a large size and are dynamically distributed throughout the mitochondrial network. Therefore, nucleoids are unlikely to move freely within mitochondrial matrix (Bonekamp and Larsson, 2018). It may be anchored at the inner membrane of mitochondria and its distribution may depend on mitochondrial fusion and fission (Elgass et al., 2013). There are evidences showing that deficiency of the large GTPase dynamin-related protein 1 (Drp1), a major regulator during mitochondrial fission, leads to remodeling of nucleoid clustering (Ban-Ishihara et al., 2013). Mitochondrial topoisomerase 3a (Top3a) also plays an essential role in genome separation and nucleoid distribution because it can deconcatenate newly replicated mtDNA (Goffart et al., 2019).

### Mitochondrial Nucleoid Modifications

The protein scaffold of mtDNA nucleoids can also be epigenetically and post-translationally modified, just like the histones in the nDNA nucleosomes. There are more than 50 nucleoid-related proteins, in which TFAM is the only protein whose behavior is highly similar with that of histones. Therefore, mitochondrial nucleoid modifications refer to TFAM modifications. Based on current studies, TFAM can be acetylated, phosphorylated and ubiquitinated, thereby affecting its function in mtDNA packaging.

#### TFAM Acetylation

A recent study showed that TFAM is lysine acetylated within its high-mobility-group box 1 (HMGB1), which reduces TFAM to interact with non-specific DNA through distinct kinetic pathways (King et al., 2018). Another study showed that TFAM was acetylated at a single lysine residue and the level of acetylation in rat liver did not change with age (Dinardo et al., 2003). SIRT3 is the deacetylase that mediates the deacetylation of K154 of TFAM (Bagul et al., 2018; Liu et al., 2018).

#### TFAM Phosphorylation

Serine phosphorylation in HMGB1 domain of TFAM also regulates mtDNA transcription by blocking TFAM to locate at the promoter sites of target genes (King et al., 2018). Extracellular signal-regulated protein kinases (ERK1/2) can mediate phosphorylation of serine 177 in TFAM, thereby downregulating mitochondrial transcription (Wang et al., 2014). The AAA + LONP1, an ssDNA-binding protein, is responsible for the degradation of DNA-free TFAM (Chen et al., 2008). When Lon binds to heavy-strand sequences upstream of light-strand promoter (LSP^HS^) or dsDNA-TFAM, its protease activity is directly blocked, resulting in TFAM stabilization (Liu et al., 2004). When TFAM is phosphorylated within its HMGB1 domain by cAMP-dependent protein kinase in mitochondria, the ability of TFAM to bind DNA is impaired and gene transcription is activated, then TFAM is degraded by LONP1 (Lu et al., 2013).

#### TFAM Ubiquitination

In addition to the modifications above, TFAM can also be ubiquitinated and its stability or sub-location may be affected. For instance, in the retina from diabetic rats, high glucose can lead to TFAM ubiquitination, which impedes its transport to the mitochondria, resulting in subnormal mtDNA transcription and mitochondria dysfunction (Santos et al., 2014).

## mtRNA Modifications

RNA modifications are present in almost all the cellular RNAs in archaeobacteria, bacteria, plants, fungi, and animals. There are almost 150 kinds of modifications that have been found in RNA (Boccaletto et al., 2018). Mitochondrial RNAs (mtRNAs) are processed from single polycistronic precursor RNAs, however, there are also multiple different types of mt-RNAs, including 2 rRNAs, 22 tRNAs, 13 mRNAs and many ncRNAs, present in the mitochondrial matrix. This suggests that the post-transcriptional mechanisms are very important for the translation, maturation, stability and assembly of mtRNAs.

A study on m^6^A patterns in the transcriptomes of *Arabidopsis* mitochondria and chloroplast reveals that more than 86% of the transcripts are m^6^A methylated. Over 350 m^6^A sites were with ∼4.6 to ∼4.9 m^6^A sites per transcript are identified in mitochondrial genome. The extent of overall m^6^A methylation in mitochondria is much higher than that in the nucleus, but lower than that in the chloroplast. However, the m^6^A motif sequences in the transcriptome of mitochondria are similar to those of the nucleus and chloroplast, which means that m^6^A motif is conserved among them. Besides, the m^6^A patterns of rRNAs and tRNAs are also similar. However, the mitochondrial and chloroplastic m^6^A patterns in mRNAs are different from those of the nucleus. Methylated transcripts in mitochondria and chloroplast are shown to be associated with rRNA, ribosomal proteins, photosystem reaction proteins, tRNA, NADH dehydrogenase and redox systems. Different organs of the leaves, flowers and roots have differential m^6^A methylation, suggesting that m^6^A methylation plays an important role during development and differentiation (Wang et al., 2017). Besides which, more than 20 m^1^A sites are also identified in mitochondrial genes via using a transcriptome-wide analysis (Zhang and Jia, 2018).

These findings unravel a new concept of mitoepitranscriptome, referring to dynamic regulation of gene expression by the modified mtRNAs. Some mutations in mtDNA or nuclear-encoded mitochondrial modification enzymes can cause RNA modification defects, which are shown to be associated with various mitochondrial diseases. mtRNA modifications in mt-mRNAs, mt-rRNAs and mt-tRNAs are suspected to play essential roles, too. Besides, ncRNAs encoded by mtDNA may also be modified as that reported in the nDNA-derived ncRNAs (Romano et al., 2018). However, reports about mt-ncRNA modifications are absent at present, because mt-ncRNAs are not well identified yet.

### Mitochondrial rRNA Modifications

The protein/DNA ratio of mitoribosomes is higher than that of all other ribosomes, indicating that mitochondria need a more stable structure to make sure that mt-rRNA is correctly scaffolded and accurately folded (Greber and Ban, 2016). Therefore, mt-rRNA modifications seem to be important for mitochondria. Currently, there are only eight different types of modifications in 10 nucleotide sites (including m^5^U429, m^4^C839, m^5^C841, m^6^_2_A936, m^6^_2_A937 in 12S rRNA and m^1^A947, Gm1145, Um1369, Gm1370 and ψ1397 in 16S rRNA) identified in mammalian mt-rRNAs (reviewed by Bohnsack and Sloan, 2018), which is lower than that of cytoplasmic and bacterial rRNAs. These sites are clustered at peptidyl transferase center (PTC) in 16S rRNA and decoding (DSC) sites in 12S rRNA, respectively, which are similar with the ribosomal modification features in bacteria and eukaryotic cytoplasm (Amunts et al., 2015).

The study of mitochondrial rRNA modification can be traced since 1970s. A study of methylation in a fungus *Neurospora crassa* shows that the mitochondrial rRNAs have 0.05–0.16 methyl groups per 100 nucleotides (Lambowitz and Luck, 1975). The 25S and 19S rRNAs of this species have methyl contents of approximately 70 and 55%, respectively (Kuriyama and Luck, 1974). Unlike highly modified cytoplasmic rRNA, mitochondrial rRNAs (15S and 21S) of the yeast *Saccharomyces cerevisiae* only contain three modified nucleotides: a pseudouridine (Ψ2918) and two 2′-O-methylated riboses (Gm2270 and Um2791) located at the peptidyl transferase center of 21S rRNA. Mrm2p, a yeast nuclear genome encoding mitochondrial protein, is required for methylating U2791 of 21S rRNA. Mrm2p belongs to a new class of three eukaryotic RNA-modifying enzymes and is the ortholog of *Escherichia coli* FtsJ/RrmJ that can methylate a nucleotide of the peptidyl transferase center of 23S rRNA (Pintard et al., 2002). Nuclear gene-encoded PET56 catalyzes the site-specific formation of 2’-O-methylguanosine on *in vitro* transcripts of both *Saccharomyces cerevisiae* mitochondrial large ribosomal RNA (21S rRNA) and *Escherichia coli* 23S rRNA. This modification is essential for the formation of functional large subunits of the mitoribosome (Sirum-Connolly and Mason, 1993).

In mitochondrial ribosomes of mammals, such as hamster, the large ribosomal subunit RNA (17S rRNA) contains UmpGmpUp, in which Um residue is methylated relatively later than Gm residue (Dubin and Taylor, 1978). The small ribosomal subunit RNA (13S rRNA) contains, on average, approximately one residue of m^4^Cp, m^5^Cp and m^5^Up, and two residues of m2^6^Ap. m^4^Cp in 13 S rRNA is homologous to its ribose-methylated congener, m^4^Cmp of bacterial 16S ribosomal RNA. Neither m^4^Cp nor m^4^Cmp exists in cell cytoplasmic ribosomal RNA (Dubin et al., 1978).

There are three rRNA 2′-*O*-methyltransferase family members RNMTL1, MRM1, and MRM2 in mammals. MRM1 and MRM2 are bacterial and yeast homologs, whereas RNMTL1 is only found in eukaryotes. They also localize to the mitochondria, especially near mtDNA nucleoids. MRM1, MRM2, and RNMTL1 are responsible for modification of G1145, U1369, and G1370 residues of human 12S rRNA, respectively (Lee and Bogenhagen, 2014). Defective MRM2 can lead to mitochondrial encephalopathy, lactic acidosis and stroke-like episodes (MELAS)-like clinical syndrome possibly through reducing of the 2′-*O*-methyl modification at specific uracil position of 12S rRNA (Garone et al., 2017).

However, mitochondrial 16S rRNA can also be methylated (m^1^A) by tRNA methyltransferase TRMT61B in all vertebrates (Bar-Yaacov et al., 2016). Pseudouridine synthase RPUSD4 plays a role in the pseudouridylation (ψ) of a single residue in the 16S rRNA, a modification that is essential for its stability and assembly into the mitochondrial ribosome (Antonicka et al., 2017). In addition to mono-methylation, the 3′ end of the rRNA of the 12S rRNA of mouse also contains two dimethylated adenines (m^6^_2_A) that are extremely highly conserved. TFB1M is a mammalian mitochondrial dimethyltransferase homologous to bacterial that is responsible for these two dimethylated adenines. Loss of TFB1M is embryonic lethal and deletion of TFB1M in heart results in complete demethylation of these two adenines of the 12S rRNA, thereby impairing mitochondrial ribosome assembly and abolishing mitochondrial translation (Metodiev et al., 2009). Besides, m^5^C911 in the mouse 12S rRNA is catalyzed by NSUN4 without forming a complex with MTERF4, which is essential in mitochondrial ribosomal biogenesis (Metodiev et al., 2014).

Modifications in mt-rRNAs are essential for their stability to ensure the normal functions of the mitoribosome. Abnormal modifications in mt-rRNAs can be associated with the dysfunction of the mitoribosome. For instance, mitochondrial mutation 1584A 12S rRNA N6, N6-dimethyladenosine (m^6^_2_A) methylation is associated with hearing loss with 1555A > G mutation (O’Sullivan et al., 2014). Lack of the two dimethylated adenines (m^6^_2_A) in 12S rRNA is associated with the pathogenesis of type 2 diabetes (Koeck et al., 2011; Sharoyko et al., 2014).

### Mitochondrial tRNA Modifications

The genetic code used during mammal mitochondrial gene expression is non-universal. Mitochondria use only 22 mt-tRNAs to decode 60 different codons. Therefore, flexible decoding is needed for mt-tRNAs. Moreover, mt-tRNA modifications are important processes for the biogenesis of the mature tRNA. Based on the current studies, mt-tRNA contains several types of modifications, such as m^1^A, m^1^G, m^2^G, m^5^C, m^3^C, τm^5^U, τm^5^s^2^U, f^5^C, Q, t^6^A, i^6^A, ms^2^i^6^A, D, m^2^_2_G and ψ (summarized by Bohnsack and Sloan, 2018), each of which is essential for biogenesis of mature mt-tRNA. These modifications are mediated by different kinds of enzymes such as MRPP1/2, PUS1, GTPBP3, MTO1, MTU1, NSUN2/3, ABH1, TRIT1/5, CDK5RAP1 and TRMT61B (Bohnsack and Sloan, 2018).

For instance, m^1^A9 can disfavor the non-functional conformation and shifts the observed equilibrium toward the functional cloverleaf (Voigts-Hoffmann et al., 2007). RNase P, a subcomplex in human mitochondria, is the endonuclease that removes tRNA 5′ extensions and is the methyltransferase responsible for m^1^G9 and m^1^A9 formation. The ability of the mitochondrial tRNA:m^1^R9 (R = G/A) methyltransferase (TRMT10C and SDR5C1) to modify both purines is uncommon among nucleic acid modification enzymes. In this process, PRORP, a short-chain dehydrogenase, is required as a partner protein (Vilardo et al., 2012).

NOP2/Sun RNA methyltransferase family member 2 (NSUN2) is an RNA methyltransferase previously shown to introduce m^5^C in tRNAs, mRNAs and microRNAs encoded by nucleic genome. However, NSUN2 can also be imported into mitochondrial matrix and introduces m5C at positions 48, 49, and 50 of several mitochondrial tRNAs, including mt-tRNA^Tyr^, mt-tRNA^His^, mt-tRNA^Leu(UUR)^, mt-tRNA^Phe^, and mt-tRNA^Glu^. However, NSUN2 inactivation does not remarkably affect mitochondrial tRNA stability and OxPhos in differentiated cells (Van Haute et al., 2019).

NSUN3, a RNA methyltransferase that localizes to mitochondria and methylates cytosine 34 (C34) at the wobble position of mt-tRNA^Met^ via specifically recognizing the anticodon stem loop (ASL) of the tRNA. Meanwhile, a dioxygenase ALKBH1/ABH1 can oxidize ^m5^C34 mt-tRNA^Met^ to yield an ^f5^C34 mt-tRNA^Met^. During translation initiation, mt-tRNA^Met^ recognizes AUG, AUA and AUU codons and mediates incorporation of methionine on these codons, whereas mt-tRNA^Met^ recognizes AUA codons and introduces methionine incorporation during elongation. In fact, mitochondrial translation factors prefer to use ^m5^C34 mt-tRNA^Met^ during translation initiation. NSUN3 or ABH1 depletion remarkably affects mitochondrial translation (Haag et al., 2016).

mt-tRNA modifications are essential for their normal functions. Modifications in the anticodon loop can expand the decoding capacity of mt-tRNAs and make sure the fidelity of translation. Core modifications can enable the structural stability of mt-tRNAs, however, this kind of modifications can also affect its recognition by aminoacyl-tRNA synthetases in some cases (Degoul et al., 1998).

### Mitochondrial mRNA Modifications

In addition to modifications in rRNAs and tRNAs, the post-transcriptional alterations in mRNA are also important for its maturation and function, as well as regulation. There are 8 H-strand-derived mitochondrial genes (COX3, ND1/2/3/4/4L, Cytb and ATP6/8) that lack translational termination codons, but can be stopped by the addition of a polyadenine (polyA) tail to a terminal U with the help of a mitochondrial PAP poly(A) polymerase (mtPAP) and a polynucleotide phosphorylase (PNPase), thereby generating sequence (UAA)_TER_A_n_ (Chang and Tong, 2012; Shokolenko and Alexeyev, 2015). Primary transcript from the H-stand has an approximately 45 nt polyA extension. However, the length of the polyA tail varies in different cell types and different transcripts (Temperley et al., 2010). PolyA in the 3′-termini can mediate the stability or instability of the transcript possibly depending on additional polyA-binding factors or sequence-specific proteins, which may affect the translation of mt-DNA-encoded genes (Rorbach and Minczuk, 2012).

Recently, a transcriptome-wide analysis revealed that other post-transcriptional modifications also exist in mitochondrial mRNAs (Bohnsack and Sloan, 2018). Intriguingly, m^1^A in the coding region of mitochondrial transcripts can block the corresponding protein translation (Zhang and Jia, 2018). In addition to methylation, specific residues in mitochondrial mRNAs can also be pseudouridylated by TRUB2/RPUSD3, thereby affecting mitochondrial protein synthesis and cell viability (Antonicka et al., 2017). These results mean that m1A and ψ in mt-mRNA are essential for the regulation of protein translation.

### Mitochondria-Derived Non-coding RNAs

**FIGURE 4:**
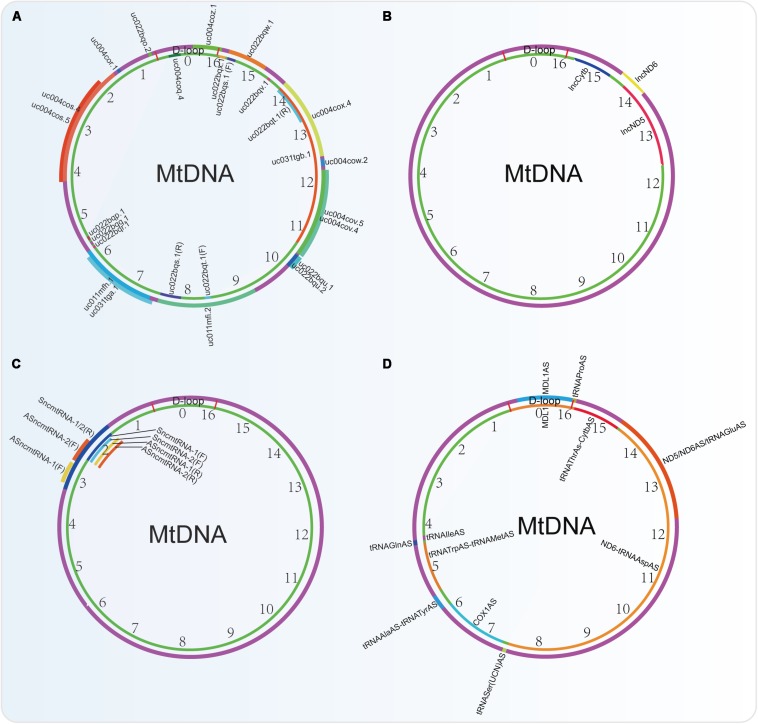
Non-coding RNAs encoded by the mtDNA identified by four different research groups. **(A)** ncRNAs obtained from the UCSC Genome Bioinformatics (2016 version). **(B)** lncRNAs identified by Rackham et al., 2011. **(C)** lncRNAs indented by researchers from Fundación Ciencia para la Vida, Chile. **(D)** ncRNAs identified by using PacBio full-length transcriptome data.

Except for mRNAs, rRNAs and tRNAs, recent reports show that mtDNA can also encode non-coding RNAs (ncRNAs), including lncRNAs and small non-coding RNAs (sncRNAs) (Table 2 and Figure 4), which are termed mtDNA-encoded lncRNAs (lncRNAs^mtDNA^) and mtDNA-encoded sncRNAs (sncRNAs^mtDNA^), respectively. However, circular RNAs (circRNAs) seem to be absent in the mitochondria (Zhang et al., 2019).

**TABLE 2:** Non-coding RNAs drived from human mtDNA.

Name	Location in mtDNA (H/L-strand)	Size (nt)	Function or relation with disease	References
uc022bqo.2	650–674, H	25	–	Kumarswamy et al., 2014; de Gonzalo-Calvo et al., 2016; Kitow et al., 2016; Thum et al., 2017; Du et al., 2018; Li et al., 2018; Schulte et al., 2019
uc004cor.1	1,603–1,634, H	32	–	
uc022bqp.1	5,543–5,566, L	24	–	
uc022bqq.1	5,585–5,606, L	22	–	
uc022bqr.1	5,690–5,714, L	25	–	
uc022bqv.1	14,674–14,698, L	25	–	
uc004cow.2	12,207–12,264, H	58	–	
uc022bqx.1	15,959–16,024, L	66	–	
uc004coq.4	235–368, L	134	–	
uc004cos.5	1,843–4,264, H	2,421	–	
uc031tga.1	5,904–7,439, H	1,535	–	
uc022bqs.1 (LIPCAR)	15504–15888, L + 7,587–7982, L	781	It predicts survival in patients with type 2 diabetes, heart failure and ST-segment elevation myocardial infarction.	
uc011mfi.2	7,585–9,206, H	1,622	–	
uc022bqt.1	8,367–8472, L + 13450–14,149, L	776	–	
uc022bqu.2	10,060–10,404, H	345	–	
uc022bqu.1	10,059–10,404, H	346	It is upregulated in patients with hypertrophic obstructive cardiomyopathy	
uc004cov.5	10,470–12,138, H	1,669	–	
uc031tgb.1	10,760–14,149, L	3,390	–	
uc004cox.4	12,908–14,149, H	1,242	It provides prognostic information for non-muscle invasive bladder cancer	
uc004cos.4	1,756–4,264, H	2,509	–	
uc022bqw.1	14,857–15,888, H	1,032	–	
uc004coz.1	15,999–16,571, H	5,73	–	
uc004cov.4	10473–12138, H	1,666	It is upregulated in patients with hypertrophic obstructive cardiomyopathy	
uc011mfh.1	5,855–7,427, H	1573	–	
lncND5	12,337–14,148, L	1,812	–	Rackham et al., 2011
lncND6	13,993–14,673, H	681	–	
lncCytb	14,747–15,887, L	1,141	–	
SncmtRNA-1 (GenBank: DQ386868.1)	1,717–2,536, L + 1,672–3,230, H	2,374	It is upregulated in the urine of patients with bladder cancer. It expresses in normal proliferating cells.	Villegas et al., 2007; Burzio et al., 2009; Rivas et al., 2012; Villota et al., 2012; Vidaurre et al., 2014; Bianchessi et al., 2015; Gao Y. et al., 2017
SncmtRNA-2 (GenBank: HM581520.1)	1,775–2,536, L + 1,672–3,230, H	2,311	It is induced by HPV-16/18-encoded in human keratinocytes. It expresses in normal proliferating cells.	
ASncmtRNA-1 (GenBank: EU863789)	2,808–3,124, H + 1,671–3,226, L	1,866	It is downregulated in the urine of patients with bladder cancer. It is downregulated in 17 types of tumor cells. binds to Dicer to recruit to the 3′-UTR of survivin mRNA, resulting in degradation of this mRNA. It is downregulated by HPV-16/18-encoded E2. It inhibits tumor growth and metastasis in the RenCa murine renal adenocarcinoma model.	
ASncmtRNA-2 (GenBank: EU863790)	2,222–2,772, H + 1,671–3,233, L	2,104	It involves in the establishment of replicative senescence by participating in the cell cycle arrest in G2/M phase, possibly through the production of hsa-miR-4485 and hsa-miR-1973. It promotes glomerular fibrosis in diabetic nephropathy via promoting the expression of pro-fibrotic factors. It is downregulated in the urine of patients with bladder cancer. It is downregulated in 17 types of tumor cells. It binds to Dicer to recruit to the 3′-UTR of survivin mRNA, resulting in degradation of this mRNA. It is downregulated by HPV-16/18-encoded E2. It inhibits tumor growth and metastasis in the RenCa murine renal adenocarcinoma model.	
tRNA^Gln^AS	4,329–4,400, H	72	–	Gao et al., 2018
tRNA^Ala^AS-tRNA^Tyr^AS	5,587–5,891, H	305	–	
tRNA^Ser(UCN)^AS	7,445–7,516, H	72	–	
ND5/ND6AS/tRNA^Glu^ AS	12,337–14,746, H	2,410	–	
tRNA^Pro^AS	15,954–16,023, H	70		
MDL1AS	16,024–407, H	953	–	
MDL1	15,956–576, L	1,192	–	
tRNA^Thr^AS-Cytb	14,747–15,953, L	1,207	–	
ND6-tRNA^Asp^AS	7,518–14,673, L	7,156	–	
COX1AS	5,901–7,442, L	1,542	–	
tRNA^Trp^AS-tRNA^Met^AS	4,402–5,579, L	1,178	–	
tRNA^Ile^AS	4,263–4,331, L	69	–	

#### lncRNAs^mtDNA^

In rat mitochondrial genome, there are some unidentified RNAs on both the H/L-strand, such as precursors of the ND2 mRNA plus the tRNA^Trp^ and the tRNAs clustered in the Ori L region. Besides them, antisense RNA species in the region of L-strand replication and D-loop region are also observed (Sbisà et al., 1992). A deep RNA sequencing study in human cardiac tissues also shows that there is a high relative abundance (71%) of lncRNAs^mtDNA^ (Yang et al., 2014). Another analysis in data sets from strand-specific deep sequencing shows that there is a significant proportion (15.02%, excluding rRNA and tRNA) of lncRNAs in the transcriptome of mitochondria in cervical cancer cell HeLa. Among them, 3 lncRNAs (lncND5, lncND6, lncCytb) transcripts are highly abundant (Figure 4B). These lncRNAs can form intermolecular duplexes and they are expressed in a cell- and tissue-specific manner. Their levels may be regulated by the nuclear-encoded proteins such as ELAC2, MRPP1, MRPP3, PTCD1, and PTCD2 (Rackham et al., 2011).

Recently, the PacBio full-length transcriptome data revealed that there are 6 lncRNAs in the H-strand and 6 lncRNAs in the L-strand of the mtDNA (Figure 4D). Among them, 2 novel lncRNAs of MDL1 and MDL1AS from the D-loop region exist ubiquitously in animal mtDNA (Gao et al., 2018).

However, from the UCSC Genome Bioinformatics (2016 version), 24 ncRNAs (including 6 microRNAs) derived from human mtDNA were provided, which are very different from those identified by the PacBio full-length transcriptome (Figure 4A). Actually, many researches that focus on the mtDNA-derived lncRNAs used this database to perform their studies. Among them, the mitochondrial lncRNA uc022bqs.1 (long intergenic non-coding RNA predicting cardiac remodeling, LIPCAR) is the best studied. This lncRNA is shown to be downregulated early after myocardial infarction but upregulated during later stages in the circulating blood of the patients (Kumarswamy et al., 2014). In addition, circulating LIPCAR also acts as a biomarker in patients with ST-segment elevation myocardial infarction (Li et al., 2018). Circulating LIPCAR is also inversely associated with diastolic function in patients with type 2 diabetes (de Gonzalo-Calvo et al., 2016). However, LIPCAR does not increase in human cardiac tissue after transcoronary ablation of septal hypertrophy, suggesting that this lncRNA is not originated from the cardiac tissues (Schulte et al., 2019). Besides, another two mtDNA-derived lnRNAs uc004cov.4 and uc022bqu.1 are also upregulated in serum of patients with hypertrophic obstructive cardiomyopathy (HOCM) but not hypertrophic non-obstructive cardiomyopathy (HNCM) (Kitow et al., 2016).

Other researchers also identified 4 lnRNAs^mtDNA^, termed SncmtRNA-1/2 and ASncmtRNA-1/2 (Figure 4C). Among them, lncRNA ASncmtRNA-2 is induced in during aging and replicative senescence in endothelial cells, but not in vascular smooth muscle cells (VSMC). Mechanically, ASncmtRNA-2 may be the non-canonical precursor of hsa-miR-4485 and hsa-miR-1973, which are originated at least in part from a mitochondrial transcript. These two microRNAs target p16 and induce a cell cycle arrest at G2M phase, thereby affecting replicative senescence establishment (Bianchessi et al., 2015). Besides, ASncmtRNA-2 is also upregulated in diabetic kidneys and high glucose-treated mesangial cells and can promote glomerular fibrosis via modulating the expression of pro-fibrotic factors in diabetic nephropathy (Gao Y. et al., 2017).

In conclusion, lncRNAs^mtDNA^ are present in the mitochondria, and may function as important epigenetic regulators for the regulation of mitochondrial function. However, a systemic study is needed to clarify the lncRNAs^mtDNA^, and their mechanisms of biogenesis and processing, as well as their functions and mode of actions.

#### sncRNAs^mtDNA^

Small non-coding RNAs (sncRNAs) are highly structured, less than 200 nt ncRNA fragments that found in bacteria, mitochondria and eukaryotes. According to their functions and characteristics, they can be classified into at least 9 types, such as microRNAs (miRNAs, ∼22 nt), Piwi-interacting RNAs (piRNA), tiny non-coding RNAs (tncRNAs), short interfering RNAs (siRNAs), repeat-associated small interfering RNAs (rasiRNAs), small modulatory RNAs (smRNAs), palindrome small RNAs (psRNAs), guide RNAs (gRNAs) and transcription initial RNAs (tiRNAs). Most of them are encoded by the nucleus genome. However, at present, mtDNA is reported to encode some sncRNAs such as miRNAs, psRNAs, tiRNAs and gRNAs. Among them, only mitochondrial-derived sncRNAs (sncRNA^mtDNA^) are widely reported.

A comprehensive analysis of the human mitochondrial transcriptome across multiple cell lines and tissues shows that sncRNAs exist in the mitochondria (Mercer et al., 2011). Remarkably, mt-tRNA^Lys^ and mt-tRNA^Me^ can be exported to the cytoplasm and bind to argonaute-2 (AGO2), an essential component of the RNA-induced silencing complex (RISC), suggesting that mt-tRNAs may act as miRNA (Maniataki and Mourelatos, 2005; Beitzinger et al., 2007). Mature tRNAs can also be cleaved by stress-activated ribonuclease angiogenin to generate 5′- and 3′-tRNA halves: a novel class of 30–40 nt small non-coding RNAs (Saikia and Hatzoglou, 2015). Besides, miR-1974, miR-1977, and miR-1978 may be encoded by the tRNA and rRNA genes in mtDNA (Bandiera et al., 2011). Some sequences of pre-miR-let7b and pre-miR-302a located in the mitochondria of human muscle can be aligned with the mtDNA, implying that these miRNAs may be derived from mtDNA (Barrey et al., 2011). These evidences suggest that the mtDNA can be a source of microRNAs (Bandiera et al., 2013).

An analysis on the mouse mtDNA identified 6 sncRNAs^mtDNA^, among which Mt-5 RNA is transcribed in antisense orientation to ND4 and Mt-6 RNA is transcribed in antisense orientation to ND6 (Lung et al., 2006). Further study shows that thousands of sncRNAs are encoded in murine and human mtDNA and most of them derived from the sense transcripts (Ro et al., 2013). The processing of sncRNAs^mtDNA^ is not only dependent on Dicer but also some mitochondrial ribonucleases that are currently unidentified (Ro et al., 2013). Overexpression of mitochondrial-derived sncRNAs can significantly enhance the expression levels of their host genes (Ro et al., 2013). Six miRNAs that are termed as miR-mit1 to miR-mit6 are identified in mouse mtDNA. Among them, miR-mit3 and miR-mit4 can target MT-RNR2 (16S rRNA) in skeletal muscles (Shinde and Bhadra, 2015). Furthermore, Mt-1 can exhibit variable length due to polyadenylation, in which contains a microRNA-like small RNA, mmu-mir-805, that is encoded in the termination association sequence (TAS) of the mtDNA, and it is upregulated in hippocampus during olfactory discrimination training in the mice (Smalheiser et al., 2011).

Besides that, mtDNA also produces psRNAs, tiRNAs (Gao et al., 2018), and gRNAs (Ochsenreiter and Hajduk, 2006). These RNAs also play essential roles in mitochondria. For instance, precise insertion and deletion of numerous uridines are required to make full-length mitochondrial mRNAs. Guide RNA responsible for COX3 mRNA can be alternatively edited to yield a stable mRNA in the mitochondria of *Trypanosoma brucei brucei* (Ochsenreiter and Hajduk, 2006).

### Nucleus-Derived Non-coding RNAs That Target mtDNA Encoded Genes

Apart from the ncRNAs^mtDNA^, nuclear DNA-derived non-coding RNAs (ncRNAs^nDNA^) including mainly lncRNAs^nDNA^ and miRNAs^nDNA^ may also mediate mitoepigenetics referring to ncRNAs^mtDNA^ that affect the translation and function of mtDNA-encoded genes. These RNAs encoded by nuclear DNA may be imported into mitochondria. In *Trypanosoma brucei*, RNA import into mitochondria has been studied well. Nuclear encoded tRNA of *T. brucei* can be imported into mitochondria (Schneider et al., 1994). In fact, *T. brucei* imports all mitochondrial tRNAs from the cytosol, and an *in vitro* study showed that there were possible some membrane-bound receptors that can mediate the import of small ribosomal RNAs (srRNAs) and tRNA in protozoon *Leishmania* mitochondria (Mahapatra et al., 1994; Mahapatra and Adhya, 1996). Subsequently, tRNA import into the kinetoplast mitochondrion of the *Leishmania tropica* were shown to be organized into a multiprotein RNA import complex (RIC) that contained 3 mitochondrion- and 8 nuclear−encoded subunits, such as Tim17 and mitochondrial heat-shock protein 70 (mtHSP70) at the inner membrane (Mukherjee et al., 2007; Tschopp et al., 2011).

However, the RNA import systems in different species exhibit some unique features (Schneider and Maréchal-Drouard, 2000). For instance, *Saccharomyces cerevisiae* imports cytoplasmic tRNA^Gln^ into mitochondria without any added protein factors (Rinehart et al., 2005). The RNA import of plant mitochondria is dependent on the voltage-dependent anion channel (Salinas et al., 2006). The study of RNA import into mammalian mitochondria is not so clear. A microarray analysis of highly purified rat liver-derived mitochondria identified 15 miRNAs^nDNA^, 5 of which were further confirmed by TaqMan 5′ nuclease assays. These miRNAs may be associated with the expression of some genes related to apoptosis, cell proliferation, and differentiation (Kren et al., 2009). Further studies also show that there are possible several ATP-dependent import pathways of nucleus-encoded RNAs to human mitochondria (Duarte et al., 2015). For example, polynucleotide phosphorylase (PNPase) is shown as a contributor to mitochondrial RNases P (MRP), 5S rRNA, tRNAs and miRNAs import into mitochondria (Wang et al., 2010; Shepherd et al., 2017). Mitochondrial enzyme rhodanese was also shown to be responsible for 5 S rRNA import into human mitochondria (Smirnov et al., 2010). Both pre-miRNAs^nDNA^ and mature miRNAs^nDNA^ were shown to be present in the human mitochondria (Barrey et al., 2011; Bandiera et al., 2013), implying that there is possible a miRNA synthesis system in the mitochondria. However, it is also reported that tRNA import into human mitochondria does not take place under normal physiological conditions, but it is possible for mutant human mitochondria to take in nucleus-encoded tRNAs (Kolesnikova et al., 2004; Mahata et al., 2006; Rubio et al., 2008). However, the RNA import to mitochondria needs to be further confirmed and its mechanism investigated.

Selected nucleus-derived ncRNAs are possibly imported into the mitochondria, where they can be involved in multiple mitochondrial biological processes to act as “messengers” between the nucleus and the mitochondria (Vendramin et al., 2017; Jeandard et al., 2019). All the microRNAs present in mitochondria are also called mitomiRNAs (mitomiRs), which is commonly used in some reports (Rippo et al., 2014; Duarte et al., 2015; Giuliani et al., 2018). Interestingly, all the mitomiRs seemed to preferentially target multiple mtDNA sites, other than nuclear-encoded mitochondrial genes, compared with a set of cytosolic miRNAs (Duarte et al., 2015).

#### lncRNAs^nDNA^ That Target mtDNA Encoded Genes

We summarize the lncRNA^nDNA^ that may affect mtDNA-encoded genes in Table 3. The behaviors of lncRNAs are multi-faced and can be implicated in various regulatory levels of gene expression, from transcription to post-translation. For instance, Cerox1 promotes the levels of mitochondrial OxPhos by upregulating mitochondrial complex I subunit transcripts through binding to microRNA-488-3p, thereby leading to a decrease of ROS production (Sirey et al., 2019). Besides, a skeletal muscle- and heart-enriched lncRNA LINC00116 can encode a highly conserved 56-AA mitoregulin (Mtln) that localizes in inner mitochondrial membrane. Mtln can interact with ND5, thereby bolstering protein complex assembly and/or stability. Overexpression of Mtln results in increasing mitochondrial membrane potential and Ca^2+^ retention capacity, decreasing fatty acid oxidation, mitochondrial ROS and matrix-free Ca^2+^, promoting respiratory complex I activity and oxygen consumption and maintaining lipid composition of the cell (Stein et al., 2018; Chugunova et al., 2019). These evidences show that lncRNAs^nDNA^ act as messengers between nDNA and mitochondria.

**TABLE 3:** Nucleus-derived long non-coding RNAs that may target mtDNA encoded genes.

Name	Target	Function	Mechanism	References
Cerox1	COX1	Decrease in reactive oxygen species and upregulation in mitochondrial oxidative phosphorylation	It binds with miR-488-3p, which can target COX1	Sirey et al., 2019
LINC00116	ND5	Perturbations in mitochondrial respiratory (super) complex formation and activity, fatty acid oxidation, tricarboxylic acid (TCA) cycle enzymes, and Ca^2+^ retention capacity	It encodes a short peptide named mitoregulin (Mtln) that can interact with ND5	Stein et al., 2018; Chugunova et al., 2019
AK055347	ATP synthase	Inhibition of cell viability of H9C2 cardiomyocytes, dysregulation of mitochondrial energy production	It downregulates ATP synthase	Chen et al., 2016

#### miRNAs^nDNA^ That Target mtDNA Encoded Genes

AGO2, a Dicer1-interacting protein that plays an essential role in short interfering RNA-mediated gene silencing, is also found to localize to mitochondria and bind to the mitochondrial transcripts COX3 and tRNA^Met^ (Bandiera et al., 2011), suggesting the activities of microRNA-mediated biological processes in the mitochondria. In addition to the miRNAs^mtDNA^, mitochondria also contain miRNAs^nDNA^. Table 4 summarizes different microRNAs^nDNA^ that can target mtDNA-encoded genes in multiple species, thereby affecting various biological or pathological processes. For instance, miR-181c was shown to target mt-COX1 in cardiomyocytes of rat (Das et al., 2012). In addition, miR-181c-5p is predicted to have 12 potential targets (12S RNA, 16S RNA, ND1, ND4, ND5, ND6, COX1, COX2, COX3, ATP6, Cytb, tRNA^Gly^) encoded by mtDNA, whereas miR-146a-5p is predicted to have 12 potential targets [16S RNA, ND1, ND2, ND4, ND5, ND6, ATP8, tRNA^Ala^, tRNA^Glu^, tRNA^Ser(UCN)^, tRNA^Ser(AGY)^] on mtRNAs (Dasgupta et al., 2015). Some microRNAs, including miR-1275, miR-1246, miR-328-5p, miR-1908, miR-1972, miR-1977, miR-638, miR-1974, miR-1978 and miR-1201, are also predicted to target mtDNA-derived RNAs (Bandiera et al., 2011). These predictions need to be further validated.

**TABLE 4:** Nucleus/mtDNA-derived MicroRNAs that have been confirmed to target mtDNA-encoded genes.

Targeted gene	MicroRNA	Species	Organism/Tissues/Cells	Biological/pathological process influenced	References
COX 1	miR-181c	*Rattus norvegicus*	Cardiomyocytes, myoblast	Mitochondrial respiration, reactive oxygen species generation	Das et al., 2012, 2017
	miR-488-3p	*Homo sapiens, Mus musculus*	Mouse Neuro-2a neuroblastoma cells, human embryonic kidney 293HEK cells	Reactive oxygen species, OxPhos	Sirey et al., 2019
	miR-2392	*Homo sapiens*	Multiple types of cancer	OxPhos, glycolysis	Fan et al., 2019
COX 2	miR-26a	*Homo sapiens*	Prostate cancer cells	Cell proliferation, apoptosis	Zhang et al., 2016
Cytb	miR-542-3p	*Homo sapiens*	Human skeletal muscle cell line (LHCN-M2)	Mitochondrial ribosomal stress, muscle wasting	Garros et al., 2017
	miR-151a-5p	*Mus musculus*	Spermatocyte cell line (GC-2)	Asthenozoospermia, mitochondrial respiratory activity	Zhou et al., 2015
	miR-2392	*Homo sapiens*	Multiple types of cancer	OxPhos, glycolysis	Fan et al., 2019
ND 2	miR-24	*Homo sapiens*	Lewis lung carcinoma (LLC) cells	Mitochondrial dysfunction, growth inhibition	Michael et al., 2017
	miR-762	*Homo sapiens*	Cardiomyocytes	Intracellular ATP levels, ROS levels, apoptosis, myocardial infarction	Yan et al., 2019
ND4	miR-2392	*Homo sapiens*	Multiple types of cancer	OxPhos, glycolysis	Fan et al., 2019
ND 4L	miR-214	*Mus musculus*	Kidney	Apoptosis, mitochondrial OxPhos	Bai et al., 2019
ND 6	miR-214	*Mus musculus*	Kidney	Apoptosis, mitochondrial OxPhos	Bai et al., 2019
ATP6	miR-378	*Mus musculus*	Cardiomyocyte cell line (HL-1)	Diabetes mellitus	Jagannathan et al., 2015
16S rRNA	miR-4485	*Homo sapiens*	Breast cancer cells	Mitochondrial complex I activity, the production of ATP, ROS levels, caspase-3/7 activation, and apoptosis	Sripada et al., 2017
	miR-mit3	*Homo sapiens*	skeletal muscles	It targets 16S rRNA	Shinde and Bhadra, 2015
	miR-mit4	*Homo sapiens*	skeletal muscles	It targets 16S rRNA	Shinde and Bhadra, 2015

However, functional analysis of miRNAs identified in highly purified rat liver-derived mitochondria showed that they were not targeted to the mitochondrial genome nor nuclear RNAs encoding mitochondrial proteins (Kren et al., 2009). This result implies that there are may be other mechanisms independent of miRNA-mediated mRNA degradation. Interestingly, microRNAs are also shown to directly affect mtRNA translation. For example, miR-1 can promote MT-ND1 and MT-CO1 translation but not their mRNA stability in an AGO2-dependent manner, thereby affecting muscle differentiation in mice (Zhang et al., 2014).

## The Roles of Mitoepigenetics in Cancer

Mitochondria are important organelles that are essential for functional eukaryotic cells. In fact, cancer cells also depend much on mitochondria. There are commonly many alterations in mitochondria induced by both extra-mitochondrial or intra-mitochondrial influencing factors that sustain excessive proliferation of cancer cells via providing energy and metabolites (Brandon et al., 2006; Wallace, 2012). For the extra-mitochondrial influencing factors, there are more than 1,000 of nucleus-encoded proteins and thousands of nucleus-encoded non-coding RNAs that can be imported into mitochondria and play a role in function, metabolism, regulation and the production, fission, fusion, trafficking and degradation of the mitochondria. Besides, many extra-mitochondrial signaling pathways such as apoptosis and mitophagy, as well as some factors can directly regulate the function of mitochondria. Intra-mitochondrial influencing factors include mutations in the mtDNA. However, most of these mutations in mtDNA do not inhibit energy metabolism in mitochondria but rather change the mitochondrial bioenergetic and biosynthetic state, which can communicate with the nucleus via modulating signaling pathways, transcriptional circuits and/or chromatin structural remodeling to meet the requirements of the cancer cells (Wallace, 2012). For instance, certain control region mitochondrial single-nucleotide variants (mtSNVs) highly co-occur with MYC oncogene amplification in prostate cancer, and predict a poorer patient survival (Hopkins et al., 2017).

Importantly, besides genetic mutations in mtDNA, mitoepigenetics is emerging as an important regulatory mode. All the mitoepigenetic networks including mtDNA methylation, mitochondrial nucleoid modifications, mtRNA methylation, mtDNA-encoded and nucleus-encoded ncRNAs, have been shown to play essential roles in tumor development and pathogenesis.

### mtDNA Methylation in Cancer

Cancer is often related with a low level of total nDNA methylation, hypermethylation of tumor suppressor gene promoters and hypomethylation of oncogene promoters (Kulis and Esteller, 2010). Therefore, it is suspected that methylation in mtDNA should be accurately modulated. The copy number of mtDNA is strictly regulated during cellular differentiation in cancer cells. In breast cancer, mtDNA methylation is maternally inherited in D-loop region, in which 8 aberrant mtDNA methylation sites are tightly dysregulated (Han et al., 2017). mtDNA copy number and ND-2 expression in colorectal cancer tissues are higher than that of the corresponding non-cancerous tissues. Methylation on the D-loop region in colorectal cancer tissues was lower than that of their corresponding non-cancerous tissues. Meanwhile, methylation on the D-loop region in stage III/IV colorectal cancer tissues is also significantly decreased, compared with that in stages I/II colorectal cancer tissues. Furthermore, D-loop region de-methylation is tightly correlated with a high mtDNA copy number and a high ND-2 expression. DNA methylation inhibitor 5-aza-deoxycytidine treatment also increases the mtDNA copy number and ND-2 expression in Caco-2 cells (Gao et al., 2015). Further study reveals that de-methylation of 4th and 6th/7th CpG islands of D-loop promoter can lead to the elevation of mtDNA copy number in colorectal cancer, thereby triggering cell proliferation, cell cycle progression and reducing apoptosis (Tong et al., 2017). These results indicate that mtDNA methylation is negatively correlated with mtDNA number and tumor progression.

Similarly, the level of 5mC at several sites of mtDNA is negatively correlated with mtDNA copy number in 143B osteosarcoma cells (Sun et al., 2018a). 5mC in mtDNA D-loop is low during tumor progression and may potentially contribute to the increase in mtDNA copy number observed in these tumor cells, including osteosarcoma and glioblastoma cells (Sun et al., 2018a). 5mC levels of D-loop also negatively correlate with ND5 and ND6 transcription during the tumorigenesis of 143B osteosarcoma cells (Sun et al., 2018a).

However, mtDNA of cancer stem-like cells is also hypermethylated and the mtDNA copy number is low, which makes them to use glycolysis for cell proliferation (Lee and St John, 2015). After sufficient mtDNA is restored in tumors to initiate tumorigenesis, 5mC in D-loop is increased to restrict further mtDNA replication (Sun et al., 2018a). That is the reason why cancer cells have a lower mtDNA copy number to maintain a “pseudo-differentiated” state and why global DNA demethylation can induce cellular differentiation and expansion of mtDNA copy number (Sun et al., 2018a).

However, there are also some studies that do not support the results above. For instance, colorectal adenomas have a low-level methylation of specific sites in mtDNA, but it is not associated with changes of mitochondrial gene transcription (Morris et al., 2018). Maekawa et al. (2004) showed that methylation of mtDNA was a rare event in the CpG sites in cancer cell lines and tissues of gastric and colorectal cancer. Hong et al. (2013) also confirmed that CpG methylation was absent in HCT116 colerectal cancer cell lines. van der Wijst et al. (2017) showed that CpG mtDNA methylation didn’t affect mtDNA gene expression, whereas 5mC in the GpC context decreased mtDNA gene transcription.

In conclusion, the relationship between mtDNA methylation and cancer should be studied further. To solve the problems, a more precise method to detect the methylated sites of methylation in mtDNA is needed. A systematic study on mtDNA methylation in both normal tissues and tumor tissues should be performed to show differences between them. Finally, the clinical significance, function and mode of actions of mtDNA methylation should be further elucidated.

In fact, these explorations will not only benefit the study of cancer biology, but also would be useful for studying other mitochondrial diseases. mtDNA methylation is also shown to be connected with various human diseases, such as Down’s syndrome and Alzheimer’s disease. The levels of mitochondrial SAM is downregulated in Down’s syndrome compared to control cells, suggesting that there is a low level of 5mC level in the mtDNA of this disease (Infantino et al., 2011). 5mC in mtDNA D-loop is observed in the blood of patients with late-onset Alzheimer’s disease patients (Stoccoro et al., 2017). Besides, 5mC in mitochondrial the transfer RNA phenylalanine (MT-TF), MT-RNR1 gene while not D-loop region is shown to be associated with metal-rich particulate matter (PM1) exposure and mtDNA copy number (Byun et al., 2013). In umbilical cord blood, 5mC in the 12S rRNA (MT-RNR1) or the D-loop control region of mtDNA is positively correlated with the level of free thyroid hormones (FT3 and FT4) and mitochondrial DNA copy numbers regulated by these two hormones (Janssen et al., 2017).

### Mitochondrial Nucleoid Modifications in Cancer

TFAM is the only nucleoid-associated protein that functions as a histone-like factor. Its expression is shown to be positively correlated with the progression of multiple cancers, including melanoma (Araujo et al., 2018), hepatocellular carcinoma (Qiao et al., 2017), non-small cell lung cancer (Xie et al., 2016), colon cancer (Lin et al., 2018), bladder cancer (Mo et al., 2013), epithelial ovarian carcinoma (Gabrielson et al., 2014), glioma (Lee et al., 2017), and breast cancer (Fan et al., 2017). These phenomena may be the results of higher mtDNA copy number that is found in tumors compared to normal tissues (Sun et al., 2018b), which makes tumor cells produce more TFAM for sufficient compaction. Otherwise, mtDNA in cancer cells may be tightly wrapped by more TFAM, which leads to a lower expression of mtDNA encoding ETC/OxPhos-related genes, thereby promoting tumor cells to use aerobic glycolysis.

TFAM can also be regulated by post-translational modifications including acetylation, phosphorylation and ubiquitination. However, there is no direct evidence showing that TFAM modifications are correlated with cancer. Since post-translational modifications of TFAM significantly affect its stability or function, and its modifications may also be tightly associated with tumor progression.

### mtRNA Methylation in Cancer

mtDNA is transcribed to produce RNA with continuous polycistrons, implying that post-transcriptional modulations are essential for RNA processing. Recently, mtRNA transcripts were shown to be differently accumulated in tumor tissues (Stewart et al., 2015). Mutation of mtRNA processing enzymes, such as ELAC2, which has RNase Z activity and functions in the maturation of mt-tRNA by removing a 3′-trailer from tRNA precursors to generate 3′ termini of tRNAs, is associated with prostate cancer incidence (Tavtigian et al., 2001). mt-tRNAs are heavily post-transcriptionally modified mtRNAs, mutations within which are also related to cancer (Brandon et al., 2006). A tRNA-dihydrouridine synthases, DUS2, which catalyzes the conversion of uridine residues to dihydrouridine in the D-loop of tRNA, is also commonly upregulated in pulmonary cancer (Kato et al., 2005). These results imply that mtRNA processing is important for cancer.

Recent studies show that mtRNA modifications, which are essential for mtRNA processing, are also major regulatory factors in tumors. In tumor tissues across 12 cancer types, there are remarkable alterations in methylation levels of m^1^A and m^1^G RNA in mitochondrial tRNAs. In normal tissues, RNA processing pathways are specifically related to mt-tRNAs methylation levels, however, these connections are lost in tumors (Idaghdour and Hodgkinson, 2017). High mt-tRNAs methylation difference predicts a poorer prognosis in a cohort of patients with kidney renal clear cell carcinoma (Idaghdour and Hodgkinson, 2017). The level of m^1^A and m^1^G methylation in mtRNAs can significantly affect mitochondria-mediated metabolism (Hodgkinson et al., 2014). In conclusions, mt-tRNAs methylation affects their maturation and thus plays emerging roles in tumorigenesis.

### ncRNAs^mtDNA^ in Cancer

There are emerging evidences showing that lncRNAs^mtDNA^ may be involved in tumorigenesis by promoting cell proliferation and tumor growth. A lncRNA with an 815 nt inverted repeat (IR) and a stem-loop structure resistant to RNase A is covalently linked to the 5′ end of 16S rRNA in human cells. It is expressed in highly normal proliferating cells while not in resting cells. The expression of this lncRNA can be induced in phytohemagglutinin (PHA)-treated resting lymphocytes, while can be reversibly blocked in aphidicolin-treat DU145 pancreatic cancer cells, in which cell proliferation is also reversibly inhibited (Villegas et al., 2007). Besides which, two antisense mtRNA transcripts that contain stem-loop structures are expressed in normal proliferating cells but significantly downregulated in tumor cells (Burzio et al., 2009). Further study shows that a family of mitochondrial ncRNAs (ncmtRNAs) with stem-loop structures can be divided into sense (SncmtRNAs) and antisense (ASncmtRNAs) members. Both of the SncmtRNAs and ASncmtRNAs are expressed in normal proliferating cells, whereas ASncmtRNAs are downregulated in various types of tumor cells. ASncmtRNAs knockdown induces cell cycle arrest and apoptosis via inhibiting survivin expression in cancer cell lines without impairing cell viability of normal cells. MicroRNAs generated by dicing of the double-stranded stem of the ASncmtRNAs can downregulate survivin. Mechanically, ASncmtRNAs binds to Dicer to recruit to the 3′-UTR of survivin mRNA, resulting in degradation of this mRNA (Vidaurre et al., 2014). Preclinical studies also show that ASncmtRNAs knockdown blocks tumor growth in melanoma and renal cancer models (Olavarria et al., 2018). Immortalization of human keratinocytes with HPV-16/18 downregulates the expression of the ASncmtRNAs and induces the expression of SncmtRNA-2. Furthermore, E6 and E7 are shown to be responsible for SncmtRNA-2 upregulation, whereas E2 oncogene is responsible for ASncmtRNAs downregulation (Villota et al., 2012).

In addition, some specific lncRNAs^mtDNA^ are highly upregulated in tumors or cancer patients’ urine and predict poor prognosis of these diseases. For instance, SncmtRNAs are upregulated and ASncmtRNAs are downregulated in the urine of patients with bladder cancer (Rivas et al., 2012). Higher level of lncRNA^mtDNA^ uc004cox.4 in urine is associated with poorer recurrence-free survival (RFS) of non-muscle invasive BC (NMIBC) and act as an independent prognostic factor for RFS of this disease (Du et al., 2018).

In conclusion, ncRNAs^mtDNA^ are important regulators during tumorigenesis and can be promising prognostic markers for cancers. Therefore, it is urgent to identify the mtDNA-encoded ncRNAs to provide a better understanding of this area.

### ncRNAs^nDNA^ That Target mtDNA Encoded Genes in Cancer

Emerging evidences also show that ncRNAs derived from nDNA also act as messengers to regulate mitochondrial function in cancer cells. For instance, lncRNA MALAT1 can be transported into mitochondria by RNA-binding protein HuR and mitochondria transmembrane protein mitochondrial carrier 2 (MTCH2) in HepG2 hepatocellular carcinoma cells. Then the 3′-fragment of this lncRNA interacts with multiple mtDNA loci, including D-loop, COX2, ND3, and CYTB. MALAT1 knockdown results in low OxPhos, reduced ATP production, inhibited mitophagy, declined mtDNA copy number, and upregulated intrinsic apoptotic pathway (Zhao et al., 2019).

In addition to lncRNAs^nDNA^, miRNAs^nDNA^ seem to play roles that are more important in epigenetic regulations of tumor cells. For instance, miR-24 targets ND2 in human Lewis lung carcinoma (LLC) cells, resulting in mitochondrial dysfunction and growth inhibition (Michael et al., 2017). miR-26a targets COX 2 in human prostate cancer cells, inhibiting cell proliferation and inducing apoptosis (Zhang et al., 2016). miR-4485 targets 16S rRNA in human breast cancer cells, leading to the decrease of mitochondrial complex I activity, the production of ATP, and inducing high ROS levels that activates caspase-3/7-dependent apoptosis (Sripada et al., 2017). miR-2392 localizes to mitochondria, silences mtDNA transcription through an AGO2-dependent mechanism, thereby inhibiting ND4, CYTB, and COX1 expression, and promotes cancer cells to chemosensitivity (Fan et al., 2019).

## Conclusion and Perspectives

The puzzle of mitoepigenetics has been uncovered gradually in recent years. New findings also significantly alter the concept of mitoepigenetics. Manev and Dzitoyeva (2013) first proposed the concept of mitoepigenetics in 2013 referring to all epigenetic regulations that are related to mitochondria. Ferreira et al. (2015) also use this concept. According to their definition, mitoepigenetics is comprised of four levels: (i) epigenetic controls of expression of nDNA-encoded mitochondrial genes; (ii) a cell-specific mtDNA content and mitochondrial activity-determined epigenetic alterations in nuclear genes expression; (iii) mtDNA variants-influenced nuclear gene expression patterns and ncDNA methylation levels; (iv) epigenetic modifications in mtDNA like 5mC and 5hmC marks. However, Manev and Dzitoyeva (2013) suggested a restricted usage of mitoepigenetics as the last ones, which also was used by subsequent researchers such as Sadakierska-Chudy et al. (2014). However, van der Wijst and Rots (2015) and Coppede and Stoccoro (2019) used a more restricted definition of mitoepigenetics as 5mC or 5hmC in mtDNA. The concept of mitoepigenetics described by Ghosh et al. (2015) includes 5mC/5hmC in mtDNA, mitochondrial modulation of nuclear DNA methylation and non-coding RNAs regulatory epigenetics in mitochondria.

**FIGURE 5:**
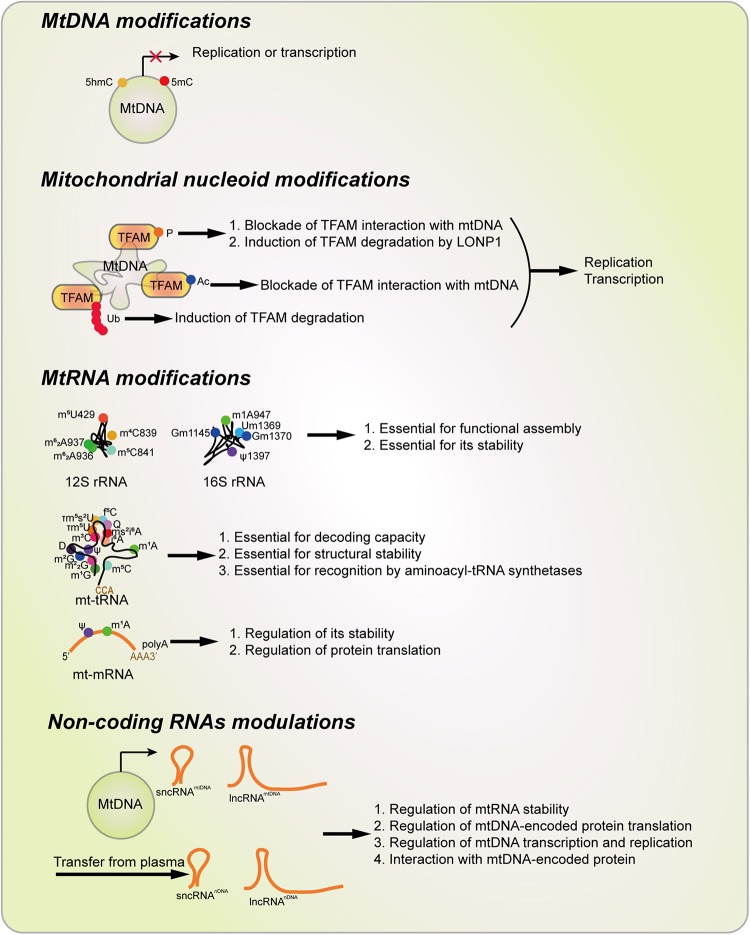
Types of Mitoepigenetics and their functions. Mitoepigenetics constitutes with four different types, including mtDNA modifications, nucleoid modifications, mtRNA modifications, and non-coding RNA modulations. Ac, acetylation; D, Dihydrouridine; f^5^C, 5-Formylcytosine; 5hmC 5-Hydroxymethylcytosine; 5mC, 5-methylcytosine; i^6^A, *N6*-Isopentenyladenosine; m^1^A, 1-Methyladenosine; m^1^G, 1-Methylguanosine; m^2^G, *N2*-Methylguanosine; m^2^_2_G, *N2,N2*-Dimethylguanosine; m^3^C, 3-Methylcytosine; m^4^C, *N4*-Methylcytosine; m^5^C, 5-Methylcytosine; m^5^U, 5-Methyluridinep; m^6^A, *N6*-Methyladenosine; m^6^_2_A, *N6,N6*-Dimethyladenosine; ms^2^i^6^A, 2-Methylthio-*N6*-isopentenyladenosine; P, phosphorylation; Ψ, Pseudouridine; Q, Queosine; t6A, *N6*-Threonylcarbamoyladenosine; TFAM, Transcription factor A, mitochondrial; τm5U, 5-Taurinomethyluridin; τm5s2U, 5-Taurinomethyl-2-thiouridine, Ub, Ubiquitination.

In this review, we definite the concept of mitoepigenetics as a study of molecular modifications occurring in mitochondria that affect mitochondrial inheritance without involving mtDNA changes. According to this definition, mitoepigenetics refers to mtDNA modifications, mitochondrial nucleoid modifications, mtRNA modifications as well as non-coding RNAs that affect the translation and function of mtDNA-encoded genes (Figure 5). This definition is narrower than the concept defined by Manev and Dzitoyeva (2013), but is an extension of the concept used by Ghosh et al. (2015) as it includes mtRNA modifications, mitochondrial nucleoid modifications and a new definition of non-coding RNAs-regulated mitoepigenetics. In fact, according to our definition, all the mitoepigenetic alterations seem to alter the expression and function of mtDNA-encoded proteins, which mainly play essential roles in ETC/OxPhos and participate in mitochondrial cellular metabolism including glucose, lipid and amino acid metabolism. Therefore, mitoepigenetics is tightly related to multiple mitochondria-mediated biological processes, such as intrinsic apoptosis (Dong et al., 2017), mitophagy (Dong and Cui, 2018; Li et al., 2019), ROS generation (Kausar et al., 2018), Ca^2+^ signaling (Bravo-Sagua et al., 2017) and hemoglobin synthesis (Fleming and Hamza, 2012).

Since dysfunctional mitochondria are tightly related to cancer initiation and cancer progression (Wallace, 2012; Vyas et al., 2016), mitoepigenetics can also involve important modulations that occur in the pathological processes of cancer. 5mC in specific sites of mtDNA seems to be decreased during tumorigenesis. This phenomenon suggests that 5mC in these sites may be prognostic markers for cancers. Besides, since recent report shows that 5hmC and 5fC contents are decreased significantly in the very early stage of HCC (Liu et al., 2019), 5hmC found in mtDNA may also make some senses during cancer initiation and progression. Besides, TFAM is also shown to be positively related to malignant progression of multiple cancers, post-translational modifications that found in this protein may also be essential modulations in cancer progression. NcRNAs derived from both the nDNA and mtDNA are also promising prognostic factors that regulate tumorigenesis. Mitoepigenetic alterations may be one of the reasons for carcinogenesis, otherwise they are results of tumorigenesis. These alterations cannot be a main reason for tumorigenesis, because cancer cells without mitochondria (ρ0 cells) still can form tumors *in vivo* (Magda et al., 2008; Sun et al., 2018b). However, epigenetic alterations in mitochondrial indeed affect the development of tumors and mitochondrial Achilles’ heel in cancer can also be targeted by mitoepigenetic modulation (Hockenbery, 2002). Anyway, these findings about the connections between cancers and mitoepigenetics may provide some new clues for the prognosis, prevention and even therapeutic strategies for these diseases.

Epigenetic regulation is a kind of reversible mode for gene expression. Until now, there are several epigenetic drugs (epi-drugs), such as 5-azacytosine, decitabine, guadecitabine, belinostat, panobinostat, vorinostat, and romidepsin are approved by FDA in the clinic to treat some diseases including cancers (Jones et al., 2016; Nebbioso et al., 2018). Besides, other epigenetic drugs such as chidamide, givinostat, quisinostat, GSK2879552 and MAK683 are on clinical trials (Berdasco and Esteller, 2019). These studies have opened a new window for the treatment of cancers. Since mitoepigenetics also plays essential roles in mitochondrial function and processing, it may open a new window for cancer therapy. However, there are some questions need to be further solved. Firstly, mtDNA methylation should be systemically studied with high-resolution methylation sequencing. Secondly, core proteins of the nucleoid should be studied further and the post-translational modifications in TFAM and their connections with cancers should be validated. Thirdly, modifications in mtRNAs including mt-rRNAs, mt-tRNAs, mt-mRNAs and ncRNAs^mtDNA^ should be further explored. Finally, both ncRNAs^mtDNA^ and ncRNAs^nDNA^ should be further characterized, their targets should be systemically identified and their clinical significances should be confirmed.

## Author Contributions

ZD wrote the manuscript, drew the figures, and made the tables. LP and HC reviewed and revised the manuscript.

## Conflict of Interest

The authors declare that the research was conducted in the absence of any commercial or financial relationships that could be construed as a potential conflict of interest.
